# Aberrant Intrinsic Activity and Connectivity in Cognitively Normal Parkinson’s Disease

**DOI:** 10.3389/fnagi.2017.00197

**Published:** 2017-06-19

**Authors:** Deborah L. Harrington, Qian Shen, Gabriel N. Castillo, J. Vincent Filoteo, Irene Litvan, Colleen Takahashi, Chelsea French

**Affiliations:** ^1^Cognitive Neuroimaging Laboratory, Research Service, VA San Diego Healthcare System, San DiegoCA, United States; ^2^Department of Radiology, University of California, San Diego, La JollaCA, United States; ^3^Movement Disorder Center, Department of Neurosciences, University of California, San Diego, La JollaCA, United States; ^4^Psychology Service, VA San Diego Healthcare System, San DiegoCA, United States; ^5^Department of Psychiatry, University of California, San Diego, La JollaCA, United States

**Keywords:** Parkinson’s disease (PD), cognition, resting-state functional MRI, functional connectivity, ALFF, ReHo

## Abstract

Disturbances in intrinsic activity during resting-state functional MRI (rsfMRI) are common in Parkinson’s disease (PD), but have largely been studied in *a priori* defined subnetworks. The cognitive significance of abnormal intrinsic activity is also poorly understood, as are abnormalities that precede the onset of mild cognitive impairment. To address these limitations, we leveraged three different analytic approaches to identify disturbances in rsfMRI metrics in 31 cognitively normal PD patients (PD-CN) and 30 healthy adults. Subjects were screened for mild cognitive impairment using the Movement Disorders Society Task Force Level II criteria. Whole-brain data-driven analytic approaches first analyzed the amplitude of low-frequency intrinsic fluctuations (ALFF) and regional homogeneity (ReHo), a measure of local connectivity amongst functionally similar regions. We then examined if regional disturbances in these metrics altered functional connectivity with other brain regions. We also investigated if abnormal rsfMRI metrics in PD-CN were related to brain atrophy and executive, visual organization, and episodic memory functioning. The results revealed abnormally increased and decreased ALFF and ReHo in PD-CN patients within the default mode network (posterior cingulate, inferior parietal cortex, parahippocampus, entorhinal cortex), sensorimotor cortex (primary motor, pre/post-central gyrus), basal ganglia (putamen, caudate), and posterior cerebellar lobule VII, which mediates cognition. For default mode network regions, we also observed a compound profile of altered ALFF and ReHo. Most regional disturbances in ALFF and ReHo were associated with strengthened long-range interactions in PD-CN, notably with regions in different networks. Stronger long-range functional connectivity in PD-CN was also partly expanded to connections that were outside the networks of the control group. Abnormally increased activity and functional connectivity appeared to have a pathological, rather than compensatory influence on cognitive abilities tested in this study. Receiver operating curve analyses demonstrated excellent sensitivity (≥90%) of rsfMRI variables in distinguishing patients from controls, but poor accuracy for brain volume and cognitive variables. Altogether these results provide new insights into the topology, cognitive relevance, and sensitivity of aberrant intrinsic activity and connectivity that precedes clinically significant cognitive impairment. Longitudinal studies are needed to determine if these neurocognitive associations presage the development of future mild cognitive impairment or dementia.

## Introduction

Cognitive symptoms in Parkinson’s disease (PD) are often overlooked despite the high risk they incur for the future development of mild cognitive impairment (MCI) or dementia (Reid et al., 2011). Even in newly diagnosed PD patients, cognitive decline may be prominent in attention and executive functions, although less frequently memory, visuospatial abilities, and language can also be affected (Muslimovic et al., 2005). Cognitive changes are associated with diverse pathologies (Gratwicke et al., 2015) and genetic factors (Lin and Wu, 2015) that may carry different prognostic significance. As treatments that slow symptom progression develop, it is vital to understand mechanisms of cognitive change before clinical symptoms manifest, since optimal interventions will depend on early detection.

In this regard, intrinsic activity during resting-state functional MRI (rsfMRI) is of keen interest, but in PD it has been largely studied in *a priori* subnetworks using seed-based methods and independent components analyses (ICA) (Baggio et al., 2015a). While these approaches have produced important insights into intrinsic functional connectivity (iFC) disturbances in PD, their cognitive significance is poorly understood, with some studies finding no association between cognitive measures and default mode network (DMN) connectivity (Gorges et al., 2015) and others reporting that weakened DMN connectivity correlates with worse memory and visual cognition (Tessitore et al., 2012; Lucas-Jimenez et al., 2016). Recently, a growing number of studies have applied complementary analytic approaches to investigate abnormal intrinsic activity throughout the whole brain, including the amplitude of low-frequency fluctuations (ALFF) and regional homogeneity (ReHo). ALFF measures the intensity of intrinsic low frequency activity (Zuo et al., 2010; Yan et al., 2013), which is proxy for the magnitude of neural activity during rest. ReHo measures the local functional synchrony of activity in a given voxel with its nearest neighboring voxels, which normally is highly coherent owing to the functional similarity of nearby clusters (Zuo et al., 2013). These metrics exhibit high test-retest reliably (Li et al., 2012; Zuo et al., 2013), rendering them advantageous for clinical imaging. Both measures can reveal aberrant activity underlying cognitive changes, since they relate to cognitive abilities in health and disease (Dai et al., 2012; Tian et al., 2012; Zhang et al., 2012; Zou et al., 2013; Long et al., 2016; Ren et al., 2016).

In PD, ALFF and ReHo are abnormal throughout the brain including the striatum (Wu et al., 2009; Hou et al., 2014; Sheng et al., 2016; Xiang et al., 2016), frontal or sensorimotor areas (Wu et al., 2009; Skidmore et al., 2013; Yang et al., 2013; Borroni et al., 2015; Hu X. F. et al., 2015; Li et al., 2016; Xiang et al., 2016), temporal, parietal, and/or occipital cortex (Wu et al., 2009; Long et al., 2012; Choe et al., 2013; Yang et al., 2013; Zhang et al., 2013; Hu X. F. et al., 2015; Luo et al., 2015; Li et al., 2016; Sheng et al., 2016), and the cerebellum (Wu et al., 2009; Skidmore et al., 2013; Sheng et al., 2014; Chen et al., 2015). While these findings suggest extensive disturbances in both metrics, considerable discrepancies exist across studies in regional abnormalities and whether ALFF and ReHo are increased or decreased in PD relative to controls. Conflicting results may be due to small PD samples (11 to ≈20) (Wu et al., 2009; Long et al., 2012; Skidmore et al., 2013; Yang et al., 2013; Borroni et al., 2015; Hu X. F. et al., 2015; Li et al., 2016) and testing patients on dopamine medication (Zhang et al., 2013; Hu X. et al., 2015; Hu X. F. et al., 2015; Lucas-Jimenez et al., 2016; Xiang et al., 2016), which alters ALFF and ReHo (Wu et al., 2009; Hou et al., 2014). Most notably, few studies have applied the Movement Disorders Society Task Force Level II criteria to screen for MCI in PD cohorts (Litvan et al., 2012). Rather, cognitive status is typically assessed using dementia screening tests, which are insensitive to MCI (Goldman et al., 2015). Thus, much of our current knowledge about the earliest changes in brain functioning in PD is likely confounded by the inclusion of patients who are already showing clinically significant cognitive impairment. The meaning of increases and decreases in various rsfMRI measures more generally is also poorly understood, as relationships to individual differences in cognitive abilities and clinical factors typically have not been examined.

The present study builds upon previous studies by characterizing disturbances in ALFF and ReHo in cognitively intact older controls and cognitively normal PD patients (PD-CN) who were screened for MCI using the Movement Disorders Society Level II criteria. We then investigated if regional disturbances in ALFF and ReHo altered iFC with regions within the same network and/or between different networks (long-range), which supports information integration across brain networks. It was hypothesized that PD-CN patients would exhibit more circumscribed regional changes in these metrics than previously reported, and that aberrant regional ALFF and ReHo would also alter long-range iFC. We predicted that executive, memory, and visual-organization abilities would correlate with rsfMRI abnormalities, particularly in frontostriatal and temporal systems (Filoteo et al., 2014; Pirogovsky-Turk et al., 2015). We also sought to determine if functional changes in PD-CN were related to brain atrophy. To determine the sensitivity of neurobehavioral markers in PD-CN, classification accuracy of rsfMRI markers was compared to volumetric and cognitive variables that differed between the groups.

## Materials and Methods

### Participants

The Institutional Review Board at the VA San Diego Healthcare System approved the study. All subjects provided signed written informed consent. The sample consisted of 31 PD patients who met the PD United Kingdom Brain Bank Criteria and 30 healthy controls. The groups did not differ in age, educational level, and premorbid intelligence (Wechsler Test of Adult Reading), but the control group contained a higher percentage of females (**Table 1**). Patients were taking dopamine agonist monotherapy (*n* = 2), levodopa monotherapy (*n* = 5), or levodopa combination therapy (*n* = 24), and were in Hoehn and Yahr stages 1 (23%), 2 (42%), and 3 (35%). Neuropsychological testing was conducted when patients were taking their dopamine medication, since withdrawal of therapy can slow responses on timed tests, thereby confounding the interpretation of cognitive changes. For MRI scanning, subjects stopped medication overnight for a minimum of 14 h. Motor symptoms were assessed using Part III of the Unified Parkinson’s Disease Rating Scale (UPDRS). **Table 1** shows that UPDRS total motor, tremor, and postural instability/gait disorder (PIGD) symptoms (Jankovic et al., 1990) were significantly greater off than on medications.

**Table 1:** Demographic, clinical, and cognitive variables.

	Parkinson’s	Control	Statistic^a^	$\eta_{p}^{2}$
Age (years)	67.4 (7.5)	68.6 (7.2)	0.46	0.01
Education (years)	17.0 (2.3)	16.5 (1.8)	0.89	0.02
Sex (% females)	29.0	63.3	7.22^∗∗^	
Handedness (% right handed)	90.3	90.0	1.20	
Wechsler Test of Adult Reading	44.6 (4.9)	45.2 (4.1)	0.26	0.00
Mini-Mental Status Exam	29.3 (0.9)	29.5 (0.7)	1.08	0.02
Hamilton Depression Scale	3.6 (2.4)	2.0 (2.7)	4.83^∗^	0.09
Epworth Sleepiness Scale	8.6 (4.1)	7.0 (2.7)	1.98	0.04
Disease duration (years)	5.4 (3.8)			
Levodopa dosage equivalence^b^	738.2 (393.7)			
UPDRS total motor ON ^c^	27.9 (13.1)			
Tremor ON	2.7 (2.1)			
PIGD ON	2.5 (2.2)			
UPDRS total motor OFF	38.1 (14.6)			
Tremor OFF	3.6 (2.4)			
PIGD OFF	3.0 (2.3)			
**Attention and working memory**				
Adaptive digit ordering	5.5 (1.1)	5.6 (1.3)	0.74	0.00
Attention subscale (MDRS)	36.15 (1.3)	36.16 (1.0)	0.04	.001
**Executive**				
Verbal fluency-letters (DKEFS)	37.9 (11.3)	46.2 (14.1)	6.34^∗^	0.10
Inhibition/switching (DKEFS)	67.9 (19.5)	63.3 (16.7)	0.74	0.01
**Memory**				
CVLT-2 long delay free recall	9.1 (3.4)	11.5 (2.9)	4.86^∗^	0.08
Logical memory II (WMS-III)	28.6 (6.9)	31.2 (8.6)	1.22	0.02
**Visuospatial**				
Judgment of Line Orientation	24.6 (4.4)	24.8 (3.3)	0.67	0.01
Hooper Visual Organization	25.5 (2.2)	25.8 (2.9)	0.96	0.02
**Language**				
Boston Naming	57.8 (2.0)	57.2 (2.4)	0.41	0.01
Similarities (WAIS-IV)	28.3 (4.2)	28.0 (5.6)	0.01	0.00

*CVLT-2, California Verbal Learning Test Version 2; DKEFS, Delis Kaplan Executive Function System; MDRS, Mattis Dementia Rating Scale; PIGD, Postural instability/gait difficulty on the UPDRS; WAIS IV, Wechsler Adult Intelligence Scale III; WMS III, Wechsler Memory Scale III; UPDRS, Unified Parkinson’s Disease Rating Scale. ^a^F and χ^2^ (sex, handedness) statistics. All *F*-tests used ANCOVA, adjusting for sex. Tabled values are unadjusted raw score means (standard deviations). ^b^Levodopa dosage equivalence was calculated using the method of Tomlinson et al. (2010). ^c^Total motor, tremor, and PIGD scores were significantly greater OFF than ON medications (*F* = 107.9, *p* < 0.00001; *F* = 18.6, *p* < 0.0002; *F* = 9.6, *p* < 0.004, respectively). Tremor and PIGD scores were computed based on Jankovic et al. (1990). ^∗^*p* < 0.05; ^∗∗^*p* < 0.01.*

Exclusion criteria included metal in the head, neurological diagnoses other than PD, psychiatric diagnoses, history of alcohol or substance abuse, positive MRI findings (e.g., infarcts, vascular disease), use of anticholinergics or cognitive medications (e.g., Donepezil), and complaints of cognitive deficits. PD volunteers with axial tremors were excluded. Patients with upper/lower limb tremors that might cause head motion when tested off medication were also excluded. Both PD and control volunteers were excluded if they met the Movement Disorders Society Level II criteria for PD-MCI (Litvan et al., 2012), which hereafter is referred to as Level II criteria. Using two tests for each of five domains (**Table 1**), MCI was defined as > 1.5 standard deviations below the control group mean on at least two tests in a single domain or different domains. Patients were also excluded if they reported problems with cognitive functioning in daily life (UPDRS Part I, item 1). Analyses of covariance (ANCOVA), adjusting for sex, showed that the PD group had lower scores than controls on the Letter Fluency and CVLT-2 long delay free recall tests. While this indicates declining verbal fluency and delayed recall at the group level, individual patients did not exhibit clinically significant cognitive decline indicative of MCI (i.e., impairment on both Letter Fluency and CVLT-2). Self-reports of daytime sleepiness (Epworth Sleepiness Scale) did not differ between the groups. Depression symptoms (Hamilton Depression Scale) were slightly greater in the PD than the control group, but within normal to mild ranges (0 to 8) for both groups. Depression scores were not correlated with levodopa dosage equivalence or disease duration (*r* = 0.09, *p* = 0.65). Disease duration, levodopa dosage equivalence, (Tomlinson et al., 2010) and Hamilton Depression Scale scores did not correlate with neuropsychological test performances.

### Neuroimaging Protocol

High resolution T1-weighted anatomic images were acquired on a GE 3T Discovery MR750 scanner using an eight-channel head coil. Foam padding limited head motion in the coil. During rsfMRI scans, subjects fixated on a cross in the center of a screen. Echo-planar images were acquired in an oblique orientation, perpendicular to the anterior–posterior commissure, to minimize susceptibility artifacts, using a single-shot, blipped, gradient-echo, EPI pulse sequence (30.5 ms TE, 2.0 s TR, 90° flip angle, 25.6 cm FOV, 64 × 64 matrix, 150 acquisitions, contiguous 4 mm slices). High-resolution T1-weighted anatomical images maximized differentiation of the white and gray matter boundary (3D spoiled gradient-recalled at steady state, minimum full TE, 7.8 ms TR, 600 TI, 8° flip angle, 1-mm slices, 25.6 cm FOV).

### rsfMRI Analysis

The maximum framewise displacement was less than 1.0 mm in all study participants. Mean framewise displacement did not differ significantly [*F*(1,59) = 0.46, *p* = 0.50, $\eta_{p}^{2}$ = 0.01] between the PD and control groups (PD: mean = 0.041 mm, *SD* = 0.023; Control: mean = 0.038 mm, *SD* = 0.016). Motion correction was performed using SLice-Oriented MOtion Correction (Beall and Lowe, 2014), which performs an in-plane slice-wise motion registration, followed by an out-of-plane motion parameter estimation and regularization. Data were then processed and analyzed using the Analysis of Functional NeuroImages (AFNI) software^1^. The first 4 volumes of the time series were discarded. Remaining volumes were time shifted, transformed to Talairach space, and spatially filtered (4 mm Gaussian kernel). Motion parameters (12 regressors for each of the six demeaned motion parameters and their six derivatives) were also regressed out of the time series at each voxel.

The analysis of ALFF was performed using 3dRSFC (Taylor and Saad, 2013). ALFF is the sum of the low-frequency spectral (0.01–0.10 Hz) amplitudes of the Fourier-transformed time series, scaled by the mean ALFF value in the brain. ReHo was calculated using Kendall’s coefficient of concordance between the time series of a given cluster and neighboring voxels (Zang et al., 2004). A ReHo value was assigned to the central voxel of every cubic cluster using a cubic cluster size of 27 voxels (3dReHo) (Taylor and Saad, 2013). For group comparisons, ALFF and ReHo data were spatially smoothed using a 6 mm full-width at half-maximum Gaussian kernel.

Areas that showed significant group differences in ALFF and ReHo were selected as seed regions of interest (ROI) for analyses of iFC, which hereafter are designated as fcALFF and fcReHo variables to underscore the source of aberrant rsfMRI associated with the seeds. In these analyses, the voxel time-series were averaged for each seed and then cross-correlation coefficients were calculated between the time series in each ROI and the rest of brain. Correlation maps were transformed to Fisher *Z* scores for group comparisons.

### Volumetric Analysis

In preliminarily analyses, group differences in regional volumes did not vary between hemispheres. Thus, bilateral cortical and subcortical volumes were analyzed. Brain volumes were derived using the automated FreeSurfer 5.3 Pipeline^2^. Volumetric measures were adjusted for total intracranial volume to account for individual differences in head size. Group comparisons were conducted for lobular cortical volumes (frontal, parietal, temporal, occipital), cerebellum, basal ganglia (putamen, caudate, globus pallidus), medial temporal structures (parahippocampus, hippocampus, entorhinal cortex, amygdala, and temporal pole), cortical white-matter, and cerebrospinal fluid (CSF).

### Statistical Analysis

Group comparisons of ALFF, ReHo, and iFC were conducted using *t*-tests (3dttest++). Voxelwise statistical thresholds were derived from 5000 Monte Carlo simulations (3dClustSim), which estimated the voxel probability and minimum cluster size threshold needed to obtain a familywise alpha of *p* < 0.05. Because spatial thresholds are biased against small activation clusters such as the basal ganglia, which was a ROI, thresholds were derived separately for basal ganglia (caudate, putamen, globus pallidus, nucleus accumbens, substantia nigra, subthalamic nucleus) and cortical (all other brain regions) volumes. A corrected alpha of *p* < 0.05 was obtained using a voxelwise probability of *p* < 0.02 and a minimum cluster size of 33 voxels for the cortex and 13 voxels for the basal ganglia. Second level ANCOVAs tested for group differences in these variables adjusting for sex. Owing to the multiple voxelwise group tests that were conducted for each fcALFF and fcReHo seed, corrected *p*-values from these analyses were further adjusted using the false discovery rate (FDR; *q* > 0.05).

Analyses of covariance (sex adjusted) was used to test for group differences in (1) cortical, cerebellar, CSF, and cortical white-matter volumes (seven variables), (2) basal ganglia volumes (three variables), and (3) medial temporal lobe volumes (five variables), applying the FDR adjustment.

Discriminant function analyses were performed on rsfMRI, volumetric, and cognitive variables that significantly differed between groups to estimate their sensitivity in distinguishing neurocognitive changes in PD-CN from controls. The purpose of these analyses was to identify variables that might be sensitive signatures of neuropathological changes in individual PD-CN patients. Outcomes from these analyses could inform the development and refinement of measures that may have the potential to serve as markers of neuropathology, which could be evaluated in future studies to determine if they track disease progression longitudinally and predict who will develop MCI. To reliably estimate classification accuracy, a bias-corrected and accelerated bootstrap (1,000 bootstrapped samples) method was used (Efron, 1987). Receiver operating curve analyses (ROC) then evaluated the goodness-of-fit of the discriminant model by analyzing the area under the curve (AUC) for the sensitivity and specificity distributions relative to the null hypothesis (AUC = 0.50). The AUC indicates the overall accuracy of a linear weighted-combination of variables in distinguishing a PD patient from healthy individuals.

Stepwise multiple regression analyses examined the correlation of aberrant rsfMRI variables in PD with performances on Verbal Fluency, CVLT-2 long delay free recall, and Hooper Visual Organization tests (FDR adjusted). These tests are representative of executive, memory and visuospatial functions, which correlate with volumes of frontostriatal, medial temporal, or ventral object-based (temporal-occipital) systems in PD without MCI or dementia (Goldman et al., 2012; Beyer et al., 2013; Filoteo et al., 2014; Pirogovsky-Turk et al., 2015). For cognitive variables, standardized age, sex, and education adjusted residuals were analyzed. For rsfMRI and volumetric measures, standardized age and sex adjusted residuals were analyzed, since education was not correlated with imaging measures. The same analyses explored the association between discriminant function scores and cognitive variables.

## Results

### Group Differences in ALFF and ReHo

**FIGURE 1:**
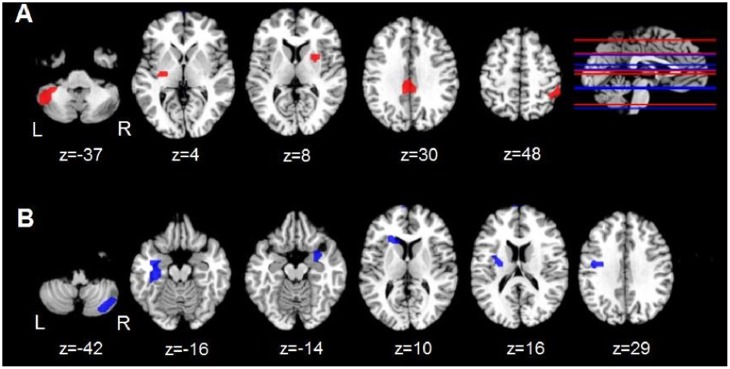
Group differences in regional ALFF and ReHo. **(A)** Axial views of regions showing aberrant ALFF (red) in the PD-CN group relative to the control group. **(B)** Axial views of regions showing aberrant ReHo (blue) in the PD group relative to the control group. The top right brain displays a sagittal view of the *z* axis associated with aberrant regional ALFF (red lines) and ReHo (blue lines). L and R = left and right hemisphere; z = superior to inferior Talairach coordinate for aberrant activity.

Relative to the control group, the PD-CN group exhibited lower ALFF in the posterior cingulate (PCC) and bilateral putamen and higher ALFF in the left posterior cerebellum and right inferior parietal cortex (**Table 2** and **Figure 1**). ReHo was lower in the PD-CN group relative to controls in the right posterior cerebellum, but higher in the left pre-central and post-central gyrus, bilateral parahippocampus/entorhinal cortex, left putamen and left caudate. Effect sizes for most group differences were large ($\eta_{p}^{2}$ ≥ 0.14).

**Table 2:** Group differences in regional ALFF and ReHo.

Brain region	*x*^a^	*y*	*z*	Volume (mm^3^)	*P*-value^b^	$\eta_{p}^{2}$
**ALFF**						
***Control > PD***						
Posterior cingulate (BA 23,31)	-2	-31	30	2752	0.0001	0.22
Right anterior putamen	26	5	8	1088	0.0003	0.20
Left posterior putamen, globus pallidus	-29	-16	4	832	0.005	0.13
***PD > Control***						
Left cerebellum (lobule VII)	-42	-48	-37	5248	0.0009	0.17
Right inferior parietal (BA 40)	47	-42	48	2560	0.01	0.10
**ReHo**						
***Control > PD***						
Right cerebellum (lobule VII)	37	-64	-42	4096	0.001	0.17
***PD > Control***						
Left pre-central and post-central (BA 6,3,4)	-38	-11	29	5568		
Right parahippocampus, entorhinal cortex	29	3	-14	2752	0.0005	0.19
Left parahippocampus	-32	-18	-16	2752	0.001	0.17
Left anterior putamen	-27	-8	16	2112	0.002	0.16
Left caudate	-19	22	10	1408	0.002	0.15

^a^*Numbers in the *x*, *y*, and *z* columns are Talairach coordinates. ^b^*P*-values for ANCOVAs tests of group differences, controlling for sex. BA, Brodmann area.*

### Group Differences in iFC of Aberrant Regional ALFF and ReHo

**FIGURE 2:**
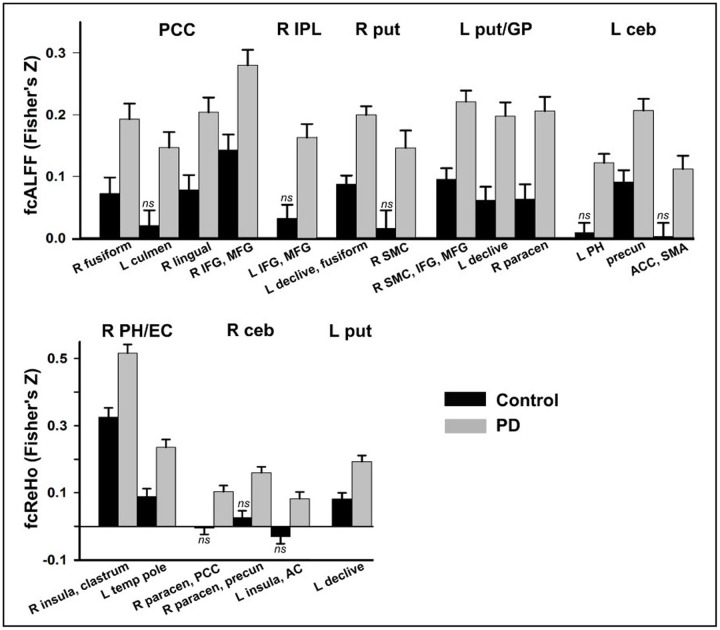
Group differences in functional connectivity. Functional connectivity of seeds (top of graphs) with other brain regions (*x* axis) is shown for the control and PD-CN groups. Fisher’s *z*-scores are plotted on the *y* axis (mean/standard error bars) for seeds that showed aberrant ALFF (fcALFF) and ReHo (fcReHO) in the PD-CN group. In the control group, functional connectivity between a seed and a region that did not differ significantly from baseline (one sample *t*-test) is designated by *ns*. AC, auditory cortex; ACC, anterior cingulate cortex; ceb, posterior cerebellum; EC, entorhinal cortex; GP, globus pallidus; L and R, left and right hemisphere; IFG, inferior frontal gyrus; IPL, inferior parietal lobule; MFG, middle frontal gyrus; paracen, paracentral; PCC, posterior cingulate cortex; PH, parahippocampus; precun, precuneus; put, putamen; SMC, sensorimotor cortices; SMA, supplementary motor area; temp pole, temporal pole.

No group differences were found in iFC for three fcReHo seeds (left pre-central/post-central gyrus, caudate, and parahippocampus). For all other seeds, iFC was significantly stronger in the PD-CN than the control group (**Figure 2**). In the control group, one-sample *t*-tests showed that iFC of some seeds did not differ significantly from baseline (designated by ‘ns’ in **Figure 2**). This result indicated an expansion of connectivity with additional brain regions in the PD-CN group. Large effect sizes were found for all group tests ($\eta_{p}^{2}$ ≥ 0.14; **Table 3**).

**Table 3:** Significant group differences in intrinsic functional connectivity.

	Region	*x*^a^	*y*	*z*	*F*^b^	Volume (mm^3^)	*P*-value^b^	$\eta_{p}^{2}$
**fcALFF Seed**								
PCC (BA 23,31)	R fusiform gyrus	30	-41	-20	10.70	3264	0.0018	0.16
	L culmen	-10	-41	-20	12.06	2816	0.0009	0.17
	R lingual gyrus	26	-73	-20	13.15	2496	0.0006	0.19
	R IFG, MFG (BA 44,45,46)	50	15	12	14.05	2432	0.0004	0.20
R inferior parietal (BA 40)	L IFG, MFG (BA 45,46)	-46	35	4	16.25	3008	0.0002	0.22
R anterior putamen	L declive, fusiform gyrus	-30	-69	-20	29.16	3520	0.000001	0.34
	R sensorimotor (BA 3,4)	30	-25	48	9.75	2432	0.0027	0.14
L posterior putamen, GP	R sensorimotor, IFG, MFG (BA 2,3,4,6)	54	15	32	23.60	5696	0.00001	0.29
	L declive	-46	-49	-28	18.34	3456	0.0001	0.24
	R paracentral (BA 5)	10	-33	48	17.29	2368	0.0001	0.23
L cerebellum (lobule VII)	L parahippocampus	-22	-1	-24	25.10	3904	0.00001	0.30
	Precuneus (BA 7)	2	-49	44	17.11	2560	0.0001	0.23
	ACC, SMA (BA 24,6)	-2	3	36	11.27	2432	0.001	0.16
**fcReHo Seed**								
R parahippocampus, EC	R insula, claustrum (BA 13)	34	-1	-20	23.71	2752	0.00001	0.29
	L temporal pole (BA 38)	-38	19	-32	18.51	2368	0.0001	0.24
R cerebellum (lobule VII)	R paracentral/PCC (BA 5,31)	2	-9	40	17.05	5120	0.0001	0.23
	R paracentral, precuneus (BA 5,7)	18	-49	60	21.87	4224	0.00002	0.27
	L insula, auditory cortex (BA 13,41,42)	-54	-13	12	14.56	3648	0.0003	0.20
L anterior putamen	L declive	-30	-69	-24	18.55	4544	0.0001	0.24

^a^*Numbers in the *x*, *y*, and *z* columns are Talairach coordinates. ^b^*F* statistics are based on ANCOVA tests for group differences, adjusting for sex. All F-tests of group differences were significant after FDR adjustment. BA, Brodmann area; L, left hemisphere; R, right hemisphere; ACC, anterior cingulate cortex; EC, entorhinal cortex; GP, globus pallidus; IFG, inferior frontal gyrus; MFG, middle frontal gyrus; PCC, posterior cingulate cortex; SMA, supplementary motor area.*

**FIGURE 3:**
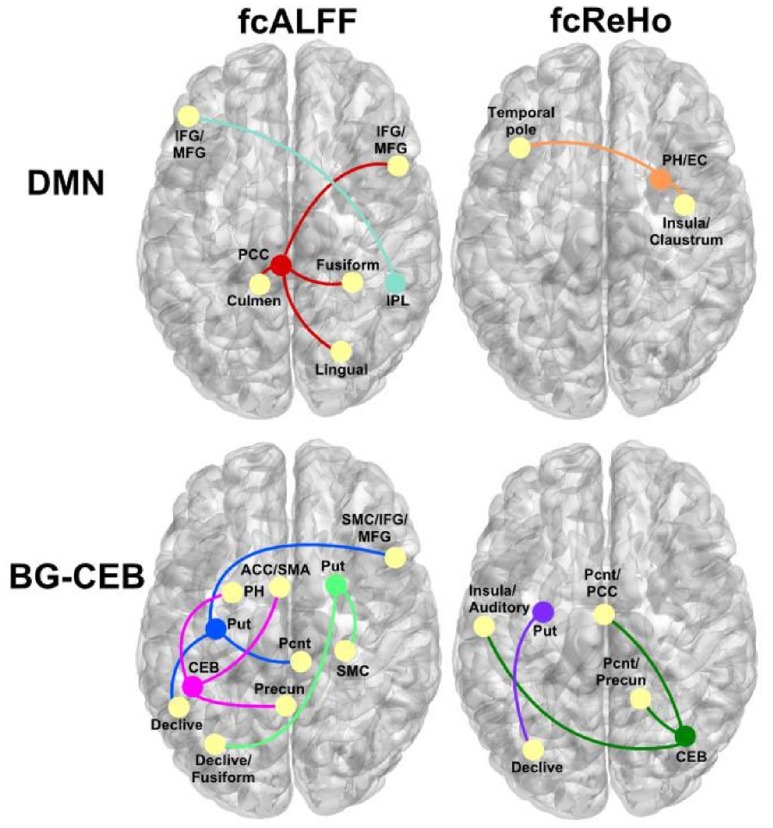
Abnormal patterns of strengthened functional connectivity in the PD-CN group for regions showing aberrant ALFF (fcALFF) and ReHo (fcReHo). *Top row*, regions of the DMN that showed stronger connectivity (colored circles/lines) with other brain regions (yellow circles). *Bottom row*, basal ganglia (BG), and posterior cerebellum (CEB) regions that showed stronger connectivity with other brain areas. ACC, anterior cingulate cortex; EC, entorhinal cortex; IFG/MFG, inferior and middle frontal gyrus; PH, parahippocampus; Pcnt, paracentral; Precun, precuneus; Put, putamen; SMA, supplementary motor area; SMC, sensorimotor cortex.

**Figure 3** illustrates the patterns of abnormally stronger iFC in the PD-CN group, which were observed for regions of the DMN and the basal ganglia and posterior cerebellum (BG-CEG). For fcALFF regions of the DMN, PCC connectivity was notably stronger with visual areas (fusiform and lingual gyrus) and frontal cognitive-control (inferior and middle frontal cortex) centers. Unlike controls, PCC connectivity in PD-NC was expanded to the anterior cerebellum, as was right inferior parietal connectivity with frontal cognitive-control centers (**Figure 2**). For a fcReHo region of the DMN, right parahippocampus/entorhinal cortex connectivity in PD-CN was stronger than in controls with an element of the salience network (insula) and an object/semantic processing (temporal pole) hub.

As for the fcALFF BG-CEB regions, **Figure 3** shows that left posterior putamen/globus pallidus iFC was stronger in patients mostly with the cerebellum, sensorimotor, and somatosensory areas. While connectivity of the right anterior putamen was stronger in PD-CN than controls with cerebellar-visual areas, iFC was expanded to right sensorimotor areas in PD-CN (**Figure 2**). Similarly, iFC of the left posterior cerebellum was stronger in patients than controls with a DMN region (precuneus), but was expanded to another region of the DMN (parahippocampus) and a key element of the salience network (anterior cingulate). For fcReHo BG-CEB regions, connectivity of the left posterior putamen with the declive was stronger in PD-CN than in controls, whereas only the PD-CN group showed significant right posterior cerebellar connectivity with medial DMN regions (paracentral, PCC, precuneus), the salience network (insula), and auditory centers.

### Group Differences in Brain Volume

The temporal pole was the only structure for which volumes were significantly smaller in the PD-CN than the control group [Mean (standard deviation) Control: 0.316 (0.042); PD-CN: 0.282 (0.033); *p* < 0.006]. However, temporal pole volume did not correlate with ALFF, ReHo, or iFC (FDR *q* > 0.05).

### Discriminant Function and ROC Analyses

**Table 4** provides summary statistics for the discriminant analyses, which were performed on six sets of rsfMRI variables that were abnormal in the PD-CN group: (1) ALFF (5 variables), (2) ReHo (6 variables), (3) ALLF and ReHo combined (11 variables), (4) fcALFF (13 variables), (5) fcReHo (6 variables), and (6) fcALFF and fcReHo combined (19 variables). Analyses were also conducted for temporal pole volume and two cognitive measures (Verbal Fluency and CVLT long-delay free recall) that differed significantly between the groups (**Table 1**). For rsfMRI variables, discriminant functions showed very good to excellent sensitivity and specificity. For ALFF, the centroid was negative in PD-CN and positive in controls, consistent with mostly lower ALFF in patients. For ReHo, fcALFF, and fcReHo functions, centroids were positive in PD-CN and negative in controls, consistent with largely increased ReHo and iFC in patients. ROC analyses demonstrated excellent classification accuracy (AUC ≥ 0.89) for rsfMRI discriminant functions, but poor classification accuracy for temporal pole volume (AUC = 0.69) and cognitive variables (AUC = 0.70).

**Table 4:** Sensitivity, specificity, and accuracy of classification.

	% Correct classification		Discriminant function centroid^a^			
Variable Set	Control	PD	Control	PD	χ^2^^b^	AUC (CI)^c^
**rs-fMRI**						
ALFF	80	87	0.95	-0.92	36.45	0.91 (0.84-0.99)
ReHo	73	81	-0.85	0.83	30.63	0.89 (0.81 -0.97)
ALFF + ReHo	90	87	1.26	-1.22	51.08	0.97 (0.93-1.0)
fcALFF	77	87	-1.06	1.03	39.77	0.94 (0.88-0.99)
fcReHo	83	84	-0.99	0.95	38.05	0.92 (0.85-0.99)
fcALFF + fcReHo	93	94	-1.47	1.42	56.74	0.99 (0.97-1.0)
**Brain volume**						
Temporal Pole	71	67	0.34	-0.35	6.84	0.69 (0.55-0.82)
**Cognitive**						
Executive, memory	57	71	0.37	-0.33	6.60	0.70 (0.57-0.83)

*^a^The centroid is the mean of the discriminant function score for each group. ^b^χ^*2*^ tests of group differences were significant for each rsfMRI discriminant function (*p* < 0.0001) and the temporal pole volume (*p* < 0.01), and cognitive (i.e., DKEFS Letter Fluency, CVLT long delay free recall; *p* < 0.04) discriminant functions. ^c^AUC tests indicate that average accuracy of classification was significantly greater than random for each rsfMRI variable set (*p* < 1.90E-7), temporal pole volume (*p* < 0.01), and the cognitive variable-set (*p* < 0.01). CI, 95% confidence interval.*

### Correlation between Imaging and Cognitive Variables in PD-CN

**FIGURE 4:**
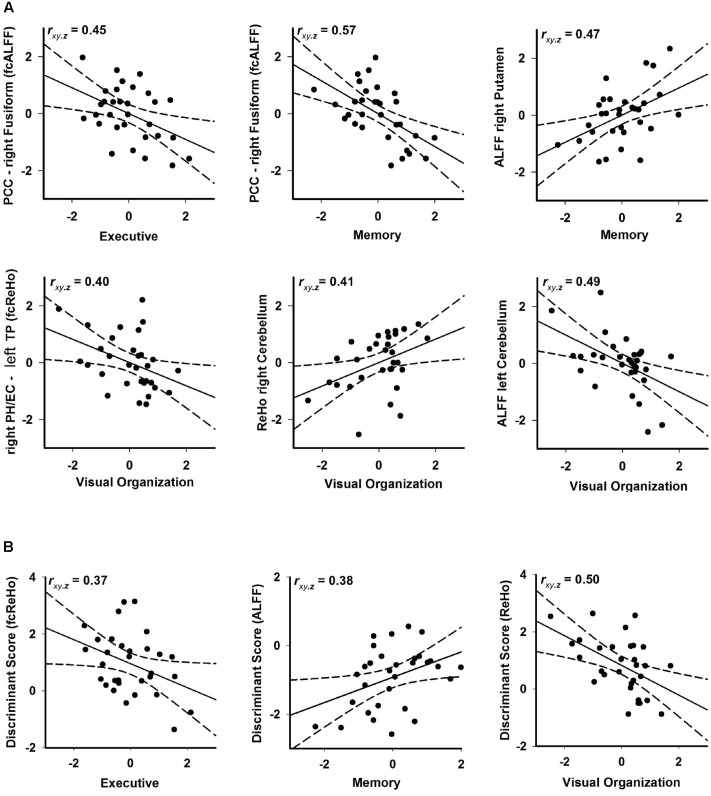
Associations between rsfMRI and executive (DKEFS Verbal Fluency), memory (CVLT-2 long delay free recall), and visual-organization (Hooper) abilities in PD-CN. **(A)** Standardized residuals are plotted for rsfMRI measures (age and sex adjusted residuals) that were significantly correlated with cognitive measures (age, sex, and education adjusted residuals). **(B)** Standardized residuals are plotted for discriminant function scores that were significantly correlated with cognitive measures. Partial correlation coefficients are displayed in the upper left corner of each graph. Solid lines show the best-fitting linear regression line and dotted lines show the 95% confidence intervals. fcALFF, functional connectivity of seeds showing aberrant ALFF; fcReHo, functional connectivity of seeds showing aberrant ReHo; PCC, posterior cingulate cortex; PH, parahippocampus; TP, temporal pole.

Stepwise regression analyses explored the best rsfMRI (ALFF, ReHo, fcALFF, fcReHo) and MRI (temporal pole volume) predictors of cognitive performances in PD-CN patients (FDR adjusted). **Figure 4A** shows that poorer executive performance was associated with increased iFC of the PCC (fcALFF seed) with the fusiform gyrus (*F* = 7.20, *p* < 0.01). Poorer memory performance was also associated increased iFC of the PCC with the fusiform gyrus (*F* = 13.77, *p* < 0.001) and decreased right putamen ALFF (*F* = 8.15, *p* < 0.008). Poorer visual-organization skills correlated with increased iFC of the right parahippocampus and entorhinal cortex (fcReHo seed) with the left temporal pole (*F* = 5.55, *p* < 0.025), decreased right cerebellar ReHo (*F* = 5.71, *p* < 0.024), and increased left cerebellar ALFF (*F* = 9.13, *p* < 0.005). No such relationships were found in the control group (*p* ≥ 0.49 for all regression analyses). Cognitive measures did not correlate with temporal pole volume (*p* ≥ 0.20 in the control group and *p* ≥ 0.30 in the PD-CN group).

The expression of discriminant function scores also predicted cognitive performances in the PD-CN group (FDR adjusted; **Figure 4B**). More aberrant fcReHo (positive), ALFF (negative), and ReHo (positive) discriminant scores were, respectively, associated with worse executive functioning (*F* = 4.56, *p* < 0.04), memory (*F* = 5.30, *p* < 0.028), and visual organization (*F* = 9.71, *p* < 0.004).

### Relationships with Clinical Variables in PD

No correlations (Pearson) were found between disease duration, levodopa dosage equivalence, Hamilton Depression Scale scores, and UPDRS motor scores (total motor, tremor, and PIGD scores on and off medication) and the rsfMRI, temporal pole volume, or cognitive variables. An exception was that higher UPDRS total motor scores off medication correlated with lower ALFF of the left putamen/GP (*r* = -0.42, *p* = 0.02).

## Discussion

The present study found that disturbances in ALFF and ReHo in PD-CN patients were more circumscribed than in studies of PD samples that were not carefully screened for MCI (Litvan et al., 2012) and thus, likely included individuals with more widespread neuropathology (Long et al., 2012; Lucas-Jimenez et al., 2016; Xiang et al., 2016) associated with MCI. Abnormal increases and decreases in ALFF and ReHo were found within the DMN, sensorimotor cortex, basal ganglia, and a posterior cerebellar lobule that mediates cognition (Stoodley et al., 2012). For regions within the DMN, we also observed a compound profile of altered ALFF and ReHo. Most regional disturbances in ALFF and ReHo were associated with strengthened long-range interactions with regions in different networks, which contrasts with the predominantly weakened iFC profiles in studies of non-demented PD that are more characteristic of advancing disease progression (Olde Dubbelink et al., 2014). Another novel finding was that abnormally stronger long-range iFC in PD-CN was partly characterized by an expansion of connectivity with additional brain regions beyond those of control subjects. Importantly, greater regional abnormalities in ALFF, ReHo, and long-range iFC were associated with worse executive, visual-organizational, or memory functioning, in support of their role in the pathogenesis of cognitive decline in PD. The results could not be attributed to disease duration, mild depression, or lingering effects of dopamine after medication withdrawal (levodopa dosage equivalence), which were not correlated with imaging or cognitive measures.

### Regional Disturbances in ALFF and ReHo in PD

Our finding of decreased ALFF in the bilateral putamen has been reported only in studies of large PD samples that were tested off medications (Hou et al., 2014; Wu et al., 2015), which underscores the effects of dopamine therapy on intrinsic activity. Moreover, increased motor symptoms on the UPDRS were selectively associated with weaker amplitude of the left posterior putamen/GP, wherein dopamine depletion is especially prominent (Bruck et al., 2006). In contrast, we found that ALFF was abnormally increased in the left posterior cerebellum, which mediates cognitive functions (Stoodley et al., 2012). Opposing patterns of basal ganglia and cerebellar ALFF are compatible with decreased striatum and increased cerebellar activation in task-activated fMRI studies of PD (Jahanshahi et al., 2010; Wu et al., 2011; Harrington et al., 2014), which is often attributed to a greater reliance on a cerebellar compensatory route for mediating behavioral states (Harrington and Jahanshahi, 2016). However, compensation should support better performance. In this regard, decreased ALFF in the right putamen was associated with worse verbal memory, consistent with striatal modulation of executive aspects of memory (Rugg et al., 2002; Filoteo et al., 2014; Pirogovsky-Turk et al., 2015). However, increased ALFF in the left posterior cerebellum correlated with poorer visual-organization abilities. Interestingly, lobule VII plays a role in mental rotation (Stoodley et al., 2012), which is engaged by the Hooper Visual Organization test. This result suggests that abnormally stronger posterior cerebellar amplitude has a pathological, rather than a compensatory, influence on visuospatial processing.

Opposing changes in ReHo were also observed in PD-CN patients, where local coherence within the left hemisphere motor circuit (posterior putamen, caudate, pre/post-central gyrus) was abnormally increased, contrary to some studies (Wu et al., 2009; Choe et al., 2013; Yang et al., 2013; Li et al., 2016; Sheng et al., 2016), but decreased in the right posterior cerebellum. These changes were not related to UPDRS total motor, tremor, or PIGD scores. However, decreased ReHo of the right cerebellum was associated with poorer visual organizational skills, possibly suggesting that reduced local coherence produces noisy communications and/or a loss in functional segregation between cerebellar-cortical circuits, which modulate higher cognitive functions (Stoodley et al., 2012). Indeed, we found a striking expansion of right posterior cerebellar connectivity with regions outside of the network of controls (**Figure 2**).

Another new finding was that PD-CN patients showed a compound profile of altered DMN activity, which may be a risk factor for MCI or dementia (Howlett et al., 2015). ALFF was decreased in the PCC and increased in right inferior parietal cortex, possibly suggesting that before cognitive symptoms manifest there is an imbalance in the functional organization of the DMN. Increased inferior parietal ALFF (Hou et al., 2014; Luo et al., 2015; Wu et al., 2015), but decreased (Wu et al., 2015) and increased PCC ALFF (Luo et al., 2015) have been reported in PD samples that were not screened for MCI. Mixed results have also been found in studies using seed-based and ICA approaches in non-demented PD and in patients that were not screened for MCI using the more rigorous Level II criteria (Tessitore et al., 2012; Gorges et al., 2015). Some of these studies reported preserved DMN connectivity (Krajcovicova et al., 2012; Rektorova et al., 2012; Seibert et al., 2012; Baggio et al., 2015b; Peraza et al., 2017), whereas others found decreased (Tessitore et al., 2012; Amboni et al., 2015; Lucas-Jimenez et al., 2016) and both increased and decreased DMN connectivity (Gorges et al., 2015). These conflicting findings may be due to small PD samples (14 to ≈ 20) (Krajcovicova et al., 2012; Rektorova et al., 2012; Seibert et al., 2012; Tessitore et al., 2012; Amboni et al., 2015; Gorges et al., 2015), testing patients on medications (Gorges et al., 2015; Lucas-Jimenez et al., 2016; Peraza et al., 2017), and less rigorous or no screening for MCI. The latter issue is relevant to our finding of preserved ALFF in frontal, occipital, and temporal cortices in PD-CN patients, which contrasts with widespread ALFF abnormalities in these regions in patients that were not screened for MCI (Long et al., 2012; Skidmore et al., 2013; Zhang et al., 2013; Hou et al., 2014; Chen et al., 2015; Luo et al., 2015; Wu et al., 2015; Xiang et al., 2016). Interestingly, we also found that ReHo was increased in PD-CN patients in another DMN region, namely the bilateral parahippocampus and right entorhinal cortex. One explanation is that abnormally *increased* regional local coherence may strengthen normal communications of a functionally similar region with other networks. Indeed, iFC of the right parahippocampus and entorhinal cortex, but also the left putamen, was aberrantly stronger in PD-CN, but only with regions within the control group network (**Figure 2**). Altogether, our results indicate that DMN abnormalities preceding MCI are characterized by compound changes in local coherence and the amplitude of intrinsic fluctuations. These results highlight the importance of leveraging different analytic approaches to more fully characterize functional disturbances in PD.

### Long-Range iFC Disturbances in PD

Regional alterations in ALFF and ReHo typically resulted in stronger iFC *between* regions in different networks, rather than *within* the same network. An exception was for increased ReHo in the pre/post-central gyrus, left caudate and left parahippocampus, which did not produce aberrant iFC profiles. Thus, increased local coherence of a region may not necessarily shape iFC with other brain regions, but when it does iFC disturbances appear to be restricted to the same networks of control subjects (**Figure 2**).

Strengthened long-range iFC of the putamen was largely found for regions within the same network as controls, except for the expansion of connectivity with the sensorimotor cortex. More striking was the expansion of posterior cerebellar iFC, which was mostly strengthened with regions in the DMN and salience network. While these findings may signify a failure to maintain regional neural coherence, the interpretation of the results is unclear since connectivity of these regions did not correlate with cognitive or clinical variables.

As for DMN regions (PCC, right inferior parietal, right parahippocampus and entorhinal cortex), iFC was notably increased with the salience network (insula) and frontal cognitive-control (IFG, MFG), visual processing (fusiform/ lingual gyrus), and object/semantic processing (temporal pose) centers in the PD-CN group. These results contrast with *decreased* PCC iFC in PD samples that were not screened for MCI (Luo et al., 2015; Lucas-Jimenez et al., 2016), which is suggestive of more advanced disease progression (Rektorova et al., 2012; Seibert et al., 2012; Olde Dubbelink et al., 2014; Amboni et al., 2015; Baggio et al., 2015b). A new finding was that aberrant long-range iFC of DMN regions were cognitively relevant, but only for those that were part of the control group network. Worse verbal fluency in PD-CN patients was associated with stronger iFC of the PCC with the fusiform gyrus, which, respectively, support executive functions (e.g., internally directed attention) and word recognition (Leech and Sharp, 2014). Poorer verbal memory was also correlated with stronger PCC - fusiform gyrus iFC, consistent with frontal mediation of executive components of memory (Rugg et al., 2002; Filoteo et al., 2014). In addition, poorer visual-organization skills correlated with stronger iFC between the right parahippocampus and the left temporal pole, which, respectively, support spatial memory and the synthesis of visual information for object identification (Pascual et al., 2015). Thus, aberrant increases in the coherence *between* the DMN and other networks had a pathological influence on cognition, which may signify difficulties in modulating DMN activity during effortful cognitive tasks (Whitfield-Gabrieli and Ford, 2012). Though other studies found no association between cognitive measures and aberrant iFC of the DMN (Gorges et al., 2015), this may be due to the small sample (*n* = 14) and the focus on disturbances *within* the DMN, which we did not find. In PD patients who were not screened for MCI using contemporary criteria, weakened iFC *within* the DMN was found that correlated with worse memory and visual abilities (Tessitore et al., 2012; Lucas-Jimenez et al., 2016), possibly suggesting that alterations within the DMN are more prominent as neuropathology progresses. Surprisingly, iFC between the right inferior parietal lobe and regions of the central executive network (left IFG, MFG) did not correlate cognitive variables in the present study. Although the reason for this finding is unclear, it is compatible with a report of stronger coherence between the DMN and right central executive network in non-demented PD, which also did not correlate with cognitive variables (Putcha et al., 2016). This pattern of connectivity was not observed in the control groups of either study, suggesting a reorganization of functional connectivity between some networks may not necessarily influence cognition or other behaviors.

### Limitations

Patients were tested after overnight withdrawal from medication to minimize the effects of dopamine therapy, which can alter and even normalize brain activation (Wu et al., 2009; Jahanshahi et al., 2010; Harrington et al., 2011; Hou et al., 2014). Although dopamine has lingering effects after short-term medication withdrawal, levodopa dosage equivalence was not correlated with rsfMRI, or cognitive variables, suggesting this factor may not have had a large effect on the results. Still, drug naïve patients are an ideal patient group to avoid the potential confounding effects of dopamine replacement therapy on brain activation. A second issue is that it is possible that more robust neurocognitive associations would be uncovered if neuropsychological testing was conducted when patients were off their medication. In particular, dopamine is known to improve some aspects of executive functioning (e.g., switching, working memory, response inhibition) that probe for functioning in frontostriatal pathways (Kehagia et al., 2010). However, medication can improve, impair, or have no effect on cognition (Gotham et al., 1988; Pillon et al., 1989; Kulisevsky et al., 1996; Lewis et al., 2005; Cools, 2006). The reasons for discrepant findings are not well understood (Robbins and Cools, 2014; Gratwicke et al., 2015), but may relate to differences among studies in tests, experimental methods, and patient characteristics (e.g., disease stage, degree of cognitive decline, levodopa dosage, and complications of dopamine therapy). For example, withdrawal of dopamine therapy may have little or no effect on neuropsychological test performance in our PD patients who were cognitively normal, since replacement therapy improves cognition (i.e., working memory) only in PD patients with poorer performance (Costa et al., 2014; Warden et al., 2016). Moreover, cognition in PD is also associated with altered functioning of non-dopaminergic neurotransmitter systems (noradrenergic, cholinergic) (Gratwicke et al., 2015). Thus, our findings that individual differences in executive, memory and visual organization skills correlated with abnormal rsfMRI measures may also be related to changes in non-dopaminergic systems. A third issue is that dopamine can alter depression symptoms (Barone et al., 2010; Kehagia et al., 2013; Caillava-Santos et al., 2015) in patients with wearing off symptoms and/or more significant signs of depression than in our PD cohort whose symptoms did not exceed the mild range. Levodopa dosage equivalence on medication is also higher in patients with than without wearing off symptoms (Kulisevsky et al., 2007) in support of role of dopamine in depression. Though some patients in our study may have experienced increased signs of depression off medication, we believe that this was not a major confound as levodopa dosage equivalence was not related to depression scores in our study. A fourth issue is that our PD-CN group had more females than our control group. However, to control for this potential confound, gender was used as a covariate and regressed out of the rsfMRI and neuropsychological data. Another issue is that iFC disturbances in PD may also exist in other cognitively relevant systems that were not studied. However, an advantage of our data-driven whole-brain analytic approach is that it motivated the selection of seeds for the iFC analyses, thereby circumventing the sometimes arbitrary selection of ROI or networks. Our approach also uncovered iFC disturbances *between* different networks or modules, which have not been widely studied using ICA approaches wherein the focus is typically on *within* network abnormalities in PD (Tessitore et al., 2012; Gorges et al., 2015; Lucas-Jimenez et al., 2016; Peraza et al., 2017).

## Conclusion and Future Directions

The present study showed that before clinically significant cognitive symptoms manifest in PD, disturbances in the brain are characterized by compound changes in intrinsic amplitude, local coherence, and long-range iFC of the DMN, basal ganglia, and the ‘cognitive’ cerebellum. Regional distributions of aberrant ALFF and ReHo differed within these systems. However, despite variations in regional patterns of increased and decreased ALFF, ReHo was increased in all of regions except the posterior cerebellum. Patterns of abnormal long-range iFC provided further insight into a potential underlying pathological mechanism. Specifically, disturbances in the magnitude of intrinsic fluctuations always increased the strength of long-range iFC in PD, whereas increased local coherence did not necessarily shape long-range iFC. The latter finding may suggest that enhanced coherence within functionally similar regions helps maintain functional segregation within brain circuits, thereby sustaining normal long-range iFC for some regions and restricting aberrant long-range iFC to circuits in the normal network. This contrasted with decreased cerebellar local coherence, which may produce a loss in functional segregation and thus, result in expanded interactions outside the normal network. It has long been thought that dopamine depletion in PD produces a loss in functional segregation within basal ganglia circuits (Pessiglione et al., 2005). One speculation is that increased regional coherence before the onset of MCI may be compensatory. However, with disease progression regional coherence may deteriorate, disrupting the specificity of interactions within brain circuits, which affects cognitive functioning. Longitudinal studies are needed to test this hypothesis and the role of other pathological mechanisms that are at play, including disturbances in the magnitude of spontaneous neural activity. These findings demonstrated the importance of exploiting different analytic approaches to more fully characterize early functional changes in PD.

In contrast to most other studies, we also found that iFC in PD-CN was characterized by disturbances in the connectivity between, rather than within networks. To our knowledge this is the first study of PD-CN to show that abnormalities in long-range iFC, especially within the DMN and cerebellum, were partly due to expanded connectivity with regions outside the control group network. However, expanded patterns of iFC were not associated with cognitive variables, possibly suggesting a loss in the fidelity of regional neural coherence or functional reorganization that does not support cognition. In this regard, we revealed for the first time that abnormal rsfMRI markers were associated in a meaningful manner with worse performances in cognitive domains that are linked to the development of MCI and dementia in PD (Muslimovic et al., 2005; Reid et al., 2011). These neurocognitive associations were notably found only for region-to-region iFC measures that were also significant in the control group. Altogether, disturbances in rsfMRI measures appeared to have a pathological, rather than a compensatory influence on cognition. Indeed, more aberrant discriminant scores, which were linear combinations of various rsfMRI markers, also correlated with worse cognitive performances. A caveat is that aberrant increases in intrinsic activity and connectivity might sustain other cognitive and behavioral functions at normal levels (Reuter-Lorenz and Park, 2014), although this possibility should be examined using a longitudinal study design. Likewise, it is possible that aberrant increases in rsfMRI markers might correlate with better performance on other behavioral measures that were not examined in the present study. In addition, individual differences may exist in neuronal compensation mechanisms, which could render relationships with cognitive variables or their absence difficult to interpret in cross-sectional analyses. Compensatory mechanisms might also postpone the onset of cognitive changes, but not necessarily correlate with individual differences in behavioral testing. Thus, longitudinal studies are needed that correlate changes in brain function with changes in cognition to better distinguish pathological from compensatory mechanisms. Nonetheless, our results underscored the excellent sensitivity of rsfMRI measures in detecting changes in PD-CN patients (≥90%), in contrast to sMRI and cognitive measures. This outcome suggests the possibility that aberrant ReHo, ALFF and associated long-range functional connectivity disturbances in PD-CN patients may be robust signatures of early neuropathological changes, some of which are correlated with performances in cognitive domains that eventually deteriorate in PD. Future studies are needed that leverage the use of other imaging modalities (e.g., diffusion weighted imaging) and analytic approaches to further develop and refine early markers of neurodegeneration that may presage the future development of MCI or dementia in PD. Longitudinal studies that track rsfMRI markers over time and their association to changes in cognition and clinical variables will be critical for elucidating the meaning of patterns of increased and decreased intrinsic activity/connectivity and their ability to track cognitive decline.

## Ethics Statement

This study was carried out in accordance with the recommendations of the Institutional Review Board at the VA San Diego Healthcare System with written informed consent from all subjects. All subjects gave written informed consent in accordance with the Declaration of Helsinki. The protocol was approved by the Institutional Review Board and the Research and Development Committee at this institution.

## Author Contributions

DH designed the study, conducted statistical analyses, interpreted the data, and assumed the lead role in writing the paper. She is the principal investigator of the primary Department of Veterans Affairs Merit Award. QS contributed to the study design, analyzed the resting-state functional MRI data, and reviewed the manuscript. GC contributed to the project design, collected the data, consulted on the analyses of neuroimaging data, and reviewed the manuscript. JF contributed to data collection, conceptual discussions about the study results, and edited important intellectual content of the paper. IL contributed to conceptual discussions about the study results and edited important intellectual content of the paper. CT collected the data, analyzed volumetric MRI data, and reviewed the manuscript. CF contributed to the statistical analyses, analyzed volumetric MRI data, and reviewed the manuscript.

## Conflict of Interest Statement

The authors declare that the research was conducted in the absence of any commercial or financial relationships that could be construed as a potential conflict of interest.

## Footnotes

1 http://afni.nimh.nih.gov

2 http://surfer.nmr.mgh.harvard.edu/
